# New Approaches to Treatment of Tricuspid Regurgitation

**DOI:** 10.3390/jcm14196878

**Published:** 2025-09-28

**Authors:** Carlo Rostagno, Alfredo Cerillo, Anna Rita Manca, Camilla Tozzetti, Pier Luigi Stefàno

**Affiliations:** 1 Dipartimento Medicina Sperimentale e Clinica, Università di Firenze, 50134 Florence, Italy; pierluigi.stefano@unifi.it; 2 Cardiochirurgia AOU Careggi, 50134 Firenze, Italy; alfredogiuseppe.cerillo@unifi.it (A.C.); annarita.manca@unifi.it (A.R.M.); 3 Medicina Interna 3 AOU Careggi, 50134 Florence, Italy; tozzettic@aou-careggi.toscana.it

**Keywords:** tricuspid valve regurgitation, tri-clip, tric-valve, endocarditis, angio-VAC 1

## Abstract

Tricuspid valve diseases are an increasing cause of cardiovascular mortality, peaking in the eighth decade of life. More than 75% of severe tricuspid regurgitations are recognized via functional mechanisms, often secondary to left heart disease and pulmonary hypertension. Surgical risk for isolated correction of tricuspid regurgitation, both repair or replacement, is associated with prohibitive risk mainly in elderly patients, with several comorbidities and right ventricular dysfunction. In the past decade, different percutaneous devices have been developed to treat a large group of high-surgical-risk patients. Early diagnosis and careful patient selection are essential to improving prognosis in severe TR. Potential treatment options may vary in different stages of disease. The current available results from present studies have proven the safety and effectiveness of these devices under proper clinical indications, although selection bias and non-randomization in most investigations at present do not allow for definite indications. Ideal anatomic and clinical parameters to predict interventional success are in continuous evolution and need definite standardization. We report three cases in which different percutaneous techniques were employed for treatment when surgery was not suitable. The literature is discussed for each condition. Despite promising results in terms of safety and success rate, further randomized studies are needed to better understand which patients may be subject to long-term effects on survival and quality of life.

## 1. Introduction

The incidence of tricuspid regurgitation (TR) in the general population is 1.2% to 1.5% [1]. The prevalence increases with age, and TR is more frequent in females. In patients aged between 70 and 83 years of age, the incidence is nearly four-fold higher in women (5.6% in women vs. 1.5% in men). Severe TR is associated with worse 1-year survival odds and unfavorable outcomes, independent of age and other comorbid conditions [2]. A recent paper reported a significant increase in age-adjusted mortality rate due to tricuspid valve disease after 2012 (6.5% annual percent change) [3]. The increasing mortality in TR may be caused by several factors. The higher number of patients with heart failure is associated with an increase in severe secondary TR [4,5], as well as the increase in right-sided devices (pacemaker, ICD). Pulmonary hypertension associated with severe pulmonary disease may contribute to this phenomenon [3].

Primary valve disease accounts for 25% of tricuspid regurgitation (TR). Anatomic valve abnormalities (congenital, myxomatous degeneration, rheumatic, neoplastic, traumatic, infective endocarditis, and endomyocardial fibrosis) are found in these patients. Damage or impingement of tricuspid valve leaflets may occur in patients undergoing right ventricle (RV) biopsy or lead implantation for pacing.

Secondary or “functional” TR is by far more common. TR due left heart valve disease, often associated with right ventricular dysfunction, often carries poor prognosis and difficult therapeutic choices. Pulmonary hypertension, right ventricular infarction, chronic right ventricular pacing, and a history of atrial fibrillation (AF) are other common causes of secondary TR [6,7,8].

The term “functional” is misleading when used for tricuspid valvular disease. Annular dilatation and/or dislocation of papillary muscles play just as important of a role in causing valve malfunction like secondary mitral valve regurgitation [9,10]. More frequent annular dilatation involves anterior and posterior tricuspid valve leaflet attachments, causing the annulus to become more circular and planar.

Geometrical abnormalities may differ between secondary TR and the so-called “idiopathic TR”, commonly related to aging and AF. Both basal RV dilatation with relatively normal RV length and marked annular dilatation but with normal tenting height of leaflets are common in idiopathic TR. Functional TR due to pulmonary hypertension causes a spherical RV deformation, with less evident annular dilation but significantly greater tenting height. These morpho-functional differences may have significant implications for treatment [11].

In TR, systolic murmur may be appreciable: it is highly variable in intensity and duration, with maximal intensity in the parasternal region at the fourth intercostal space, and occasionally is loudest in the subxiphoid area. In advanced stages of the disease, signs of congestive heart failure are pre-eminent (jugular vein distention, hepatomegaly, edema and ascites). Echocardiography, both TTE and TEE, may allow anatomic evaluation of the valve to define the severity and the pathogenetic mechanism of tricuspid regurgitation TR (severe dilation of the annulus and RV suggest secondary TR). RV function may be evaluated. The maximal velocity of the TR jet added to the central venous pressure gives a good estimate of pulmonary artery pressure. As well as for mitral regurgitation, quantitative evaluation of the severity of TR can be assessed by measuring vena contracta, regurgitant volume, and effective regurgitant area quantified by Doppler. Prolapse of the tricuspid valve caused by myxomatous degeneration as well as Ebstein’s abnormality may be evident on echocardiography. In this paper we report three paradigmatic cases treated with new percutaneous transcatheter repair devices due to prohibitive surgical risk [12]. The techniques used are discussed according to more recent literature.

## 2. Case Reports

### 2.1. Case 1

**Figure 1:**
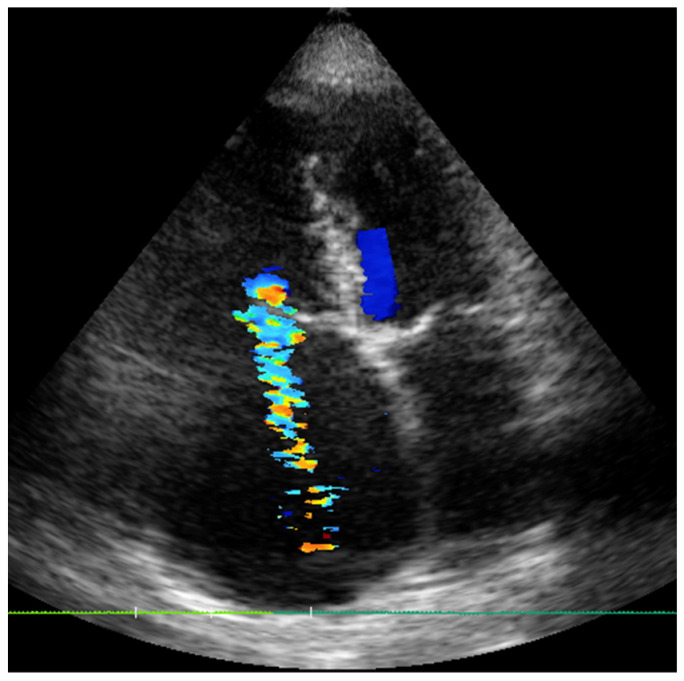
Severe TR with pulmonary hypertension, severe dilation of right atrium and ventricle.

An 82-year-old woman with a history of tobacco exposure, arterial hypertension, type 2 diabetes mellitus, atrial fibrillation on new oral anticoagulants, chronic kidney disease, and breast cancer with uneventful follow-up. She was affected by severe tricuspid regurgitation (EROA 48 mm^2^, regurgitant volume 53 mL), with two hospitalizations for heart failure in less than 1 year. Echocardiography showed severe left and right atrial dilatation, a dilated right ventricle (RVD1 48), with a TAPSE of 14 mm, pulmonary artery systolic pressure of 55 mmHg, and preserved left ventricular function (Figure 1).

The inferior vena cava was dilated (24 mm) with limited respiratory excursions. The TRISCORE was 8. The transesophageal echocardiography showed a favorable anatomy for T-TEER: a 4 mm coaptation gap and the localization of the main jet in the anteroseptal coaptation line (GLIDE score 1). Considering the patient’s age, frailty, medical history, and anatomical characteristics, the Heart Team indicated tricuspid TEER, which was performed by implanting two Triclip G4 XTW devices (Abbott, Santa Clara, CA, USA). The procedure was performed without complications, and the postoperative echocardiography showed a significant reduction in the tricuspid regurgitation, estimated as mild–moderate. Results persisted at three months follow-up.

### 2.2. Case 2

**Figure 2:**
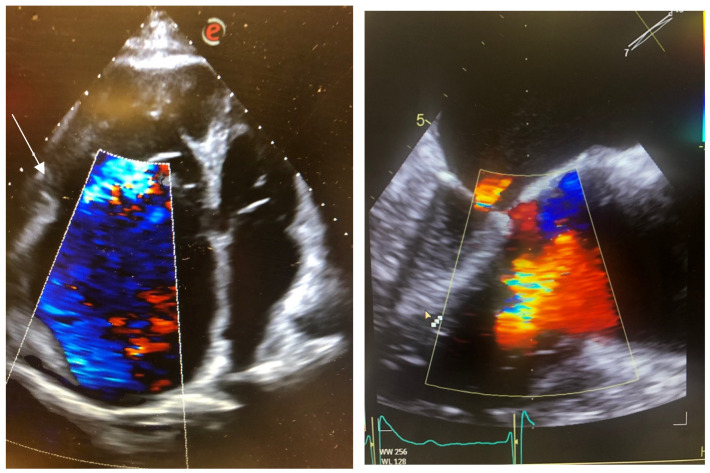
Apical 4-chamber TTE (**left**) and mid esophageal TEE (**right**) of Ebstein’s disease with giant right atrium and low tricuspid insertion (withe arrow) with a wide coaptation gap.

The second patient was a 70-year-old male affected by Ebstein’s disease with severely dilated right atrium associated with permanent atrial fibrillation on new oral anticoagulants. His cardiovascular risk factors included active smoking (12 pack-years) and arterial hypertension. He was admitted to our department for respiratory failure due to pneumococcal pneumonia, complicated by acute renal failure (creatinine peak 3.7 mg/dl). The echocardiography showed marked dilation of the right chambers (RVD1 78 mm), preserved right ventricular function (TAPSE 20 mm), severe tricuspid regurgitation with Coanda effect, a dilated inferior vena cava (26 mm) without respiratory excursions, and preserved left ventricular ejection fraction. The transesophageal echocardiography showed a severely dilated right atrium, with a thrombus of 6 × 12 mm, and severe tricuspid regurgitation with the main jet in the postero-septal coaptation line with a coaptation gap of 1.2 cm (Figure 2).

Despite the relatively young age, due to severe impairment of clinical conditions with pronounced frailty, he was not considered eligible for surgery (TRISCORE 9). Based on anatomical features (GLIDE score 3), the Heart Team identified heterotopic TTVR as the most suitable treatment for the valvular disease, once clinical stability had been reached. The patient underwent implantation of the TricValve transcatheter bicaval valve system (Products + Features, Wien, Austria) without complications.

He reported significant improvement of symptoms and quality of life at 1-year follow-up. Hepatic and renal function markers were found within normal limits.

### 2.3. Case 3

**Figure 3:**
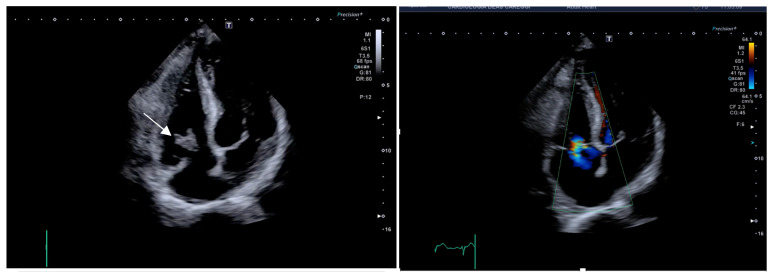
TTE, with the (**left**) panel demonstrating large vegetation of the tricuspid valve (white arrow) and the (**right**) showing mild tricuspid regurgitation after the removal procedure.

The third patient was a 70-year-old female who had undergone multiple major surgeries for neoplastic disease and was a carrier of a right brachial (PICC) for parenteral nutrition. The patient was admitted for persistent fever >38 °C. The blood cultures were positive for multi-resistant S. aureus. Transthoracic echocardiography showed an 18 × 11 mm vegetation on the tricuspid valve, causing mild regurgitation. CT scan showed findings consistent with pulmonary embolization. Moreover, abdominal recurrence of leiomyosarcoma was found. Despite antibiotic treatment (vancomycin and thereafter linezolid), fever and positive blood cultures persisted. Echocardiography showed an increase in the size of the vegetation (28 × 15 mm). (Figure 3). Considering the clinical history and the high surgical risk (TRISCORE 6, Euroscore 20%), the Heart Team of our hospital proposed an attempt of vegetation removal with the veno-venous circulation system AngioVAC (AngioDynamics, Latham, NY, USA) associated with long-term antibiotic treatment. The patient underwent the procedure without complications; the intraoperative echocardiography showed complete removal of the vegetation and a mild tricuspid regurgitation. The blood cultures turned negative on the fourth postoperative day, the inflammatory markers progressively decreased, and the patient remained afebrile. Four weeks later she underwent surgical treatment for abdominal recurrence of leiomyosarcoma and at 1-year follow-up, she was in good clinical condition.

## 3. Discussion

A conservative approach to TR was common in XX century. The hypothesis was that correction of the left-sided valve disease might result in a significant decrease or of secondary TR. Clinical experience, however, showed clearly that TR rarely changed significantly after surgery. Incomplete or late surgical correction of mitral and/or aortic valve disease is sometimes associated with a further worsening of TR. Moreover, isolated severe TR may occur also in patients without left ventricular dysfunction after either mitral valve annuloplasty or replacement. Atrial fibrillation may significantly contribute to anatomic changes, leading to tricuspid annular dilatation creating a vicious cycle that increases the degree of regurgitation.

Right ventricular dysfunction increases both surgical and 1-year mortality (from 5% to 11% and from 8% to 22%, respectively) [13]. Persisting TR is associated with a minor relief of symptoms and an impaired cardiac output response to exercise after correction of valvular diseases.

Although it has been suggested that functional TR can be ignored in patients with a predictable decrease in pulmonary resistance, there is no reliable predictive method to evaluate TR reversibility after correction of left heart valve abnormalities. Moreover, the quantification of the degree of TR suffers from methodological limits, with low reliability and repeatability. Clinical assessment may add information to echocardiography. Finally, there is no satisfactory method to assess true right ventricular function.

TR is often a challenging dilemma. Even severe TR may be tolerated for many years and managed conservatively. Nevertheless, when right heart failure and ascites develop, it is often too late for surgical correction due to unacceptable operative mortality. Moreover, the likelihood of functional recovery is poor.

Recent ACC/AHA guidelines give Class I indication for isolated tricuspid valve surgery [14]; however, randomized controlled trial (RCT) data to support surgical treatment (level of evidence C) do not exist. Class IIa (level of evidence A) has been reported for transcatheter TV treatment to improve quality of life and RV remodeling in high-risk patients in optimal medical treatment with persistently symptomatic severe TR. Patients with precapillary pulmonary hypertension and right ventricular failure should not be considered for treatment.

A decrease in right ventricle afterload after left-sided valve lesion surgery may lead to an unpredictable improvement of severe TR [9]. Because adding tricuspid valve repair during left-sided surgery does not significantly change the risks of surgery, guidelines suggest repairing tricuspid valve in patients undergoing left-sided valve surgery [13]. Right ventricular dysfunction is however associated with postoperative low cardiac output syndrome and increased early mortality. In the last decade, percutaneous transcatheter repair and replacement devices have been developed to treat high-surgical risk TR patients. An early diagnosis and referral for treatment are essential to improve results of treatment, as well as a better understanding of the different stages of disease and potential treatment options, including percutaneous options. Severe TR should be considered for treatment, irrespective of symptoms, before irreversible right ventricular failure.

The use of a clip in the tricuspid position (actually approved devices are TriClip—Abbott Vascular, Santa Clara, CA, USA—and the PASCAL device—Edwards Lifesciences, Irvine, CA, USA) gave controversial results. First experiences reported a 50% reduction in effective regurgitant orifice area, associated with 6 min walking distance. The TRILUMINATE study is a non-blind international, prospective multicenter study which compared T-TEER the TriClip implant with placebo in patients with symptomatic moderate or severe TR [15]. Follow-up at three years showed a decrease in TR severity to moderate or less in 79% of patients. Ninety-two percent of subjects had at least a one-grade reduction in TR, results similar to those found after the first year post-procedure. TR reduction was associated with improved NYHA functional class and quality of life compared to baseline and to a significant (75%) decrease in hospitalization rate. The absence of randomization did not allow us to draw conclusions about the relation between the decrease in TR regurgitation and main clinical endpoints. The cross-over allowed after the first year of follow-up is a further confounding factor. Finally, it must be outlined that only the small TriClip NT (Abbott) device was used and not the currently available TriClipG4 System (Abbott), with both longer and wider Tri- Clip XT and XTW clips, as well as independent leaflet grasping functionality to optimize clip placement.

The Tri.Fr Randomized Clinical Trial [16] included 300 patients with a mean age of 78 years and a large prevalence of females (63.7%). In total, 152 were allocated to T-TEER Valve Repair (TriClip Transcatheter Tricuspid System) + OMT and 148 to OMT alone. The primary outcome was a composite clinical end point comprising change in New York Heart Association (NYHA), class change in patient global assessment (PGA), or occurrence of major cardiovascular events. At 1 year, 109 patients (74.1%) in the T-TEER + OMT group had an improved composite score compared with 58 patients (40.6%) in the OMT-alone group. Less than 7% of patients in the T-TEER + OMT group had massive or torrential tricuspid regurgitation in comparison to 53.5% of those the OMT group (*p* < 0.001).

Recently, results from the prospective EuroTR (European Registry of Transcatheter Repair for Tricuspid Regurgitation) registry were published [17]. Patients were grouped into early, intermediate, and advanced disease. Disease stage was based on left and right ventricular function, renal function, and natriuretic peptide levels (Table 1).

Overall, among 1885 patients, 395 (21%) were categorized as early, 1173 patients (62%) as intermediate, and 317 patients (17%) as advanced disease stage. Patients with intermediate disease stage, according to the reported score system, showed a significant decrease in mortality at 1 year (OR = 0.78, 95% CI 0.52–0.99), while patients with early and advanced disease did not show mortality differences between interventional and conservative treatment. Moreover, the authors reported worse residual TR grades more frequently after T-TEER in patients who presented with mo85re advanced TR disease.

A metanalysis by Suc et al. [18] including 25 studies compared results of surgical or percutaneous treatment in comparison to optimal medical therapy in patients with isolated TR. In the study of 9108 patients, 5702 were treated with OMT (mean age 72 years), 1416 patients were treated percutaneously (mean age 71.3 years), and 1990 patients were managed surgically (mean age 59.3 years). In patients in OMT, survival after 1 year, 2 years, and 5 years were 86%, 80%, and 54%, respectively. Among patients who received a percutaneous device, in-hospital mortality was 1%, while 1- and 2-year survival were 82% and 78%. In the group who underwent surgery, in-hospital mortality was 8%. Survival was 85%, 80%, and 70%, respectively, at 1-year, 2-years and 5-years post-surgery. Etiology TR, age of patients, and severity of disease was different in the three groups of treatment. Moreover, it must be underscored that in the studies regarding percutaneous treatment, most patients were considered inoperable or at very high surgical risk [19], with some studies also reporting compassionate use [20]. Although this study suggests a high mortality rate in severe TR independent from treatment, only ongoing randomized studies with careful patient selection will provide clear indications.

Anatomic characteristics of tricuspid valve should be carefully assessed in patients suitable for T-TEER to decrease the risk of residual tricuspid regurgitation. The GLIDE score that included five parameters (septolateral coaptation gap, chordal structure density, TR jet location, en face TR jet morphology, and image quality) allowed us to predict success of treatment, defined by TR reduction ≥2 grades [21]. Other TEE parameters, such as septal–lateral leaflet to anulus-index (SL-LAI) and the recently developed AP-LAI derived from the sum of the anterior and posterior leaflets, normalized for the antero-posterior annulus diameter, are useful in predicting T-TEER results [22]. As for the percutaneous treatment of severe functional mitral regurgitation, the gap between the feasibility of the procedure and the demonstration of clinical effectiveness should pass through carefully conducted studies with standardized selection criteria and clear end points and duration for follow-up.

Other transcatheter therapies targeting TR have been tested in feasibility trials. Among these, the Trialign system (Mitralign, Tewksbury, MA, USA) or Cardioband (Edwards Lifesciences, Irvine, CA, USA) mimic the surgical Kay annuloplasty via a pair of pledgeted sutures delivered percutaneously through the right internal jugular vein. Despite some initial favorable results [23,24,25], the complexity of the procedure in comparison to T-TEER limited its clinical application.

Unfavorable anatomic abnormalities (severely dilated tricuspid annulus, and excessive coaptation gap) are associated with a high risk of failure and coaptation devices, annuloplasty devices, and transcatheter valve replacement may be unsuitable. TRICVALVE (P&F Products & Features, Vienna, Austria), a transcatheter heterotopic bicaval valves system including two self-expanding biological valves in the inferior and superior vena may be proposed for patients with relevant caval reflux, who are intractable with surgery. Implantation of TRICVALVE has less procedural complexity and usually does not need TEE monitoring and general anesthesia. Device embolization is a rare complication of transcatheter structural heart interventions [26]. The TRICUS (Safety and Efficacy of the TricValve^®^ Transcatheter Bicaval Valves System in the Superior and Inferior Vena Cava in Patients With Severe Tricuspid Regurgitation) and TRICUS EURO studies were prospective, nonblinded, nonrandomized, single-arm trials. They evaluated the TricValve system in NYHA functional Class III or IV severe TR patients with significant caval backflow in OMT and who were ineligible for open heart surgery. In total, 44 patients were included in the study, with a mean age of 76.2 ± 7.5 years, and 81.0% were women. Hepatic vein flow was achieved and persisted in 63.8%. Forty-two patients showed an improvement in 12-item Kansas City Cardiomyopathy Questionnaire score, improvement to NYHA functional Class I or II, or an increase ≥40 m in the 6 min walk test. Three patients died (6.8%) at 1-year follow-up (1 cardiovascular). Rehospitalization was 30% [27]. Another small series reported no procedure complications in eight patients who underwent TRIC valve implantation [28].

In patients with tricuspid valve endocarditis, percutaneous mechanical vegetation remotion with an Angio-Vac system appears to offer an alternative to the surgical approach in patients at high surgical risk [29]. The Angio-Vac suction has been approved by the Food and Drug Association since 2014 for the removal of thrombi in the iliocaval, pulmonary, or upper extremity vasculature. Recently, this approach has been used also for the right-sided infective endocarditis.

Debulking tricuspid valve vegetations aims to reduce bacterial load to allow antimicrobial therapy to cure the infection or to stabilize the patient as a bridge to surgery.

Most of the treated patients were i.v. drug addicts. Thrombus removal from the right side of the heart was successful in 82% of a series of 56 patients [30]. Hematoma in puncture site and retroperitoneal bleed complicated 12% of procedures. Removal of vegetation from tricuspid valve is associated with a risk close to 10% of worsening TV regurgitation needing TV replacement close to 10% [31].

According to a retrospective study, the length of hospital-stay, the need for blood transfusion, and days for therapy were significantly lower in patients who underwent Angio-Vac approach compared to the traditional TV surgery [32].

In the report from Starck et al. [33], 61 patients with right-sided endocarditis were treated with Angio-VAC. Most had a pacemaker and ICD lead vegetations and only one had tricuspid valve endocarditis. Overall success rate was >90%, including the curing of patients with MSSA sepsis and tricuspid vegetation.

These results, although promising, should be supported by larger studies. In patients with tricuspid valve endocarditis and failure of medical therapy, it will be important to prospectively evaluate the effect of Angio-VAC on mid- and long-term survival, as well as endocarditis recurrence.

## 4. Conclusions

Different percutaneous approaches have been proposed in the last decade for treatment of TR or tricuspid endocarditis. Results from current studies suggest that success rate and risk of complications are acceptable and a higher number of patients with severe tricuspid disease at high risk for conventional surgery may be treated with an improvement in symptoms and functional capacity. Nevertheless, postprocedural mortality is still high, and in some studies is not significantly different from OMT. An accurate selection of patients, in particular for T-TEER, may allow us to improve survival, although further randomized studies are needed to confirm these results.

## Figures and Tables

**Table 1:** Classification of TR severity adopted in Euri TR modified from Schlotter et al. [17].

Score	1 Point	2 Points	3 Points
LVEF (%)	≥50	40–49	<40
TAPSE (mm)	>17	13–17	<13
eGFR (mL/min/1.73 m^2^)	>60	30–60	<30
NT pro BNP or BNP (pg/mL)	<125 or 35	125–1249 or 35–349	>1250 or >350

Early disease stage: 4–6 points. Intermediate disease stage: 7–9 points. Advanced disease stage: 10–12 points.

## Data Availability

Reported data are available in the electronic records of AOU-Careggi.

## Abbreviations

The following abbreviations are used in this manuscript:

TR	Tricuspid regurgitation
OMT	Optimal medical therapy
